# *Pseudomonas spp*. are key players in agricultural biogas substrate degradation

**DOI:** 10.1038/s41598-019-49313-8

**Published:** 2019-09-06

**Authors:** Christian Buettner, Martin von Bergen, Nico Jehmlich, Matthias Noll

**Affiliations:** 1 Coburg University of Applied Sciences and Arts, Institute for Bioanalysis, Friedrich-Streib-Str. 2, 96450 Coburg, Germany; 2 Helmholtz-Centre for Environmental Research – UFZ GmbH, Department of Molecular Systems Biology, Permoserstraße 15, 04318 Leipzig, Germany; 3 University of Leipzig, Institute for Biochemistry, Brüderstraße 34, 04103 Leipzig, Germany

**Keywords:** Environmental microbiology, Environmental microbiology

## Abstract

Anaerobic degradation (AD) of heterogeneous agricultural substrates is a complex process involving a diverse microbial community. While microbial community composition of a variety of biogas plants (BPs) is well described, little is known about metabolic processes and microbial interaction patterns. Here, we analyzed 16 large-scale BPs using metaproteomics. All metabolic steps of AD were observed in the metaproteome, and multivariate analyses indicated that they were shaped by temperature, pH, volatile fatty acid content and substrate types. Biogas plants could be subdivided into hydrogenotrophic, acetoclastic or a mixture of both methanogenic pathways based on their process parameters, taxonomic and functional metaproteome. Network analyses showed large differences in metabolic and microbial interaction patterns. Both, number of interactions and interaction partners were highly dependent on the prevalent methanogenic pathway for most species. Nevertheless, we observed a highly conserved metabolism of different abundant *Pseudomonas spp*. for all BPs indicating a key role during AD in carbohydrate hydrolysis irrespectively of variabilities in substrate input and process parameters. Thus, *Pseudomonas spp*. are of high importance for robust and versatile AD food webs, which highlight a large variety of downstream metabolic processes for their respective methanogenic pathways.

## Introduction

Climate change and rising energy consumption trigger innovation in the global energy market. New approaches and expansion of existing renewable energy technologies such as biogas, solar, water and wind are needed to facilitate goals in reducing carbon dioxide emissions^1^. In contrast to other renewable energy sources, microbial biogas production is based on anaerobic fermentation of a wide variety of substrates. Different members of a microbial community accomplish the basic steps of anaerobic degradation (AD), which are hydrolysis, acidogenesis, acetogenesis and finally methanogenesis^2–4^. Hydrogenotrophic, acetoclastic and/or methylotrophic methanogenesis occur in biogas plants (BPs) alone or in combination with each other, mainly depending on abiotic parameters such as substrate, pH, temperature, ammonia content or reactor type^5–10^.

The majority of BPs in Germany are fed mainly with agricultural products such as corn, grass and few other crop plants, as well as manure and other agricultural waste. Although food residues are less common, they are of economic interest as these substrates are propitious alternative substrates or substrate additions. Therefore, microbial communities in BPs have to overcome variations of each process parameter and substrate by metabolic readjustment to enable a stable and high biogas production. Hence, detailed knowledge of involved microorganisms as well as their metabolic processes is crucial for future optimization of AD.

In previous studies, mainly species composition and abundances of BPs were characterized on 16S rRNA gene level^11–16^, which allows phylogenetic affiliation followed by predicting their potential metabolic functions. Metatranscriptomics or metaproteomics are capable tools to link present transcript or gene expression level to both, metabolic functions and phylogenetic affiliations in complex microbial community compositions^17^. Recently, high metabolic activity of members of the kingdom *Archaea* in a BP were obtained by 16S rRNA gene amplicon and metatranscriptomics approach^18^ or a combination of metagenome and metaproteome analyses^19^. Metaproteome analyses of different BPs also provided important information how different process parameters shape proteomic profiles^5^.

Methodologies for analyzing and interpreting omics data have rapidly changed in the last decade. Tools for network analyses such as MENA^20^, SparCC^21^ or CoNet^22^ are frequently used to predict interactions between microorganisms. Such network calculations are mainly based on 16S rRNA gene amplicon abundance data and therefore anaerobic degradation-based findings have to be interpreted with caution, as metabolic functions are difficult to predict. Other tools like STRING^23^ focus on discovery of protein-protein interaction networks for explanation of microbial interactions. Unfortunately, the relatively low number of reliable database entries limits those tools for AD.

In contrast to most other studies, we analyzed not only the metaproteome of a single BP but sixteen large-scale BPs. Furthermore, we measured the metaproteome of five independent replicates for each BP (same time point) to produce robust results. Main goals of this study are (i) to identify most important parameters driving the AD on protein level, (ii) to group the BPs according to their metaproteome (taxonomic and functional profiles), (iii) to arrange the BPs corresponding to their prevalent methanogenesis pathway and (iv) to identify microbial key players and their interaction patterns by a metabolic and microbial network analysis. The overall aim was to gain a better understanding of the metabolic processes during AD with a focus on methanogenesis as well as explore possibilities of metaproteomics for practical applications.

## Results

### Methanogenic proteins are dominating in most biogas plants

In total, 5,854 protein groups from 2,178 different species were identified in all BPs, whereof between 3,238 (BP01) to 3,624 (BP11) were found in average in each BP. About 77% of the identified protein groups were affiliated to Bacteria and 23% to Archaea (for community composition on different levels see Supplementary Table S1), which were assigned to 4,894 protein groups (about 84%) and their corresponding molecular function(s) (K numbers) according to KEGG^24–26^ (Kyoto Encyclopedia of Genes and Genomes).

**Figure 1:**
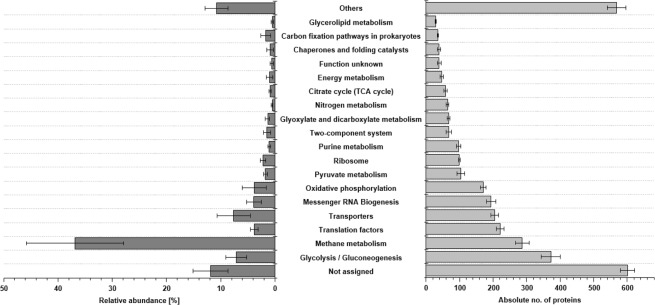
Overview of the most important KEGG pathways (TOP20) according to their absolute number of proteins (light grey, right) and their relative abundances (dark grey, left). Given are mean values over all biogas plants. Error bars indicating standard deviation over all plants. Other pathways are summarized by “others”.

In average 372 ± 28 proteins groups were assigned to KEGG pathways glycolysis/gluconeogenesis, followed by methane metabolism (287 ± 21), translation factors (220 ± 12), transporters (205 ± 12) and messenger RNA biogenesis (194 ± 14). However, highest relative protein abundances were observed for methane metabolism (36.9 ± 8.9%), transporters (7.6 ± 3.0%), glycolysis/gluconeogenesis (7.1 ± 1.9%), messenger RNA biogenesis (3.9 ± 1.4%) and translation factors (3.9 ± 0.7%) (Fig. 1). Most abundant proteins were Methyl-Coenzyme M reductase (MCR) subunit alpha with an average abundance of 7.4 ± 3.1%, MCR subunit gamma (7.1 ± 3.6%), MCR subunit beta (5.6 ± 1.4%), as well as 5,10-methylenetetrahydromethanopterin reductase with 4.1 ± 2.5% (Table 1). Other highly abundant proteins, which are not directly affiliated to methanogenesis were 60 kDa chaperonin (GroEL) (3.3 ± 1.3%), elongation factor Tu (2.8 ± 0.6%) or glyceraldehyde-3-phosphate dehydrogenase with 1.7 ± 0.5% (Supplementary Table S1).

**Table 1:** Overview of relative abundances for all KEGG orthology terms/enzyme classes associated with one or more steps of methanogenesis.

	KO	Enzyme(s)		Gene name(s)	EC-Number(s)	TOP3 most abundant organisms			C1				C2	C3	C3	C4	C5	C6	C7	C7	C8	C9	C9	C9
									Hydro	Hydro	Hydro	Hydro	Both	Hydro	Hydro	Aceto	Hydro	Aceto	Aceto	Aceto	Hydro	Aceto	Both	Both
						AcNe	BoNe	HyNe	BP06	BP07	BP08	BP12	BP15	BP10	BP13	BP01	BP16	BP05	BP02	BP03	BP04	BP14	BP09	BP11
Aceto-clastic	K00925	Acetate kinase	—	ackA	2.7.2.1	19, 1210, 1777	13, 19, 163	13, 19, 163	0.218	0.233	0.228	0.268	0.149	0.204	0.215	0.063	0.120	0.148	0.492	0.515	0.145	0.349	0.309	0.269
	K00625	Phosphate acetylt-ransferase	—	pta	2.3.1.8	19, 22, 163	19, 34, 163	19, 34, 163	0.097	0.083	0.077	0.106	0.078	0.040	0.054	0.015	0.031	0.189	0.632	0.630	0.090	0.448	0.546	0.244
	K01895	Acetyl-coenzyme A synthetase	—	ACSS	6.2.1.1	1, 9, 36	1, 9, 302	1, 9, 302	0.116	0.071	0.062	0.077	0.105	0.118	0.092	5.218	0.043	4.268	0.155	0.061	0.056	0.977	0.056	0.066
	K00193	Acetyl-CoA decarbonylase/synthase complex	Subunit Beta	cdh	2.3.1.169	1, 19, 65	1, 19, 65	1, 19, 65	0.434	0.310	0.255	0.705	0.201	0.275	0.314	1.622	0.099	2.574	3.578	3.325	0.165	3.146	1.783	1.961
	K00197		Subunit Gamma		2.1.1.245	1, 22, 65	22, 65, 163	22, 34, 163	0.824	0.861	0.788	0.829	1.219	0.437	0.951	1.673	0.229	2.344	3.862	3.516	0.537	2.890	2.049	1.690
	K00194		Subunit Delta						0.414	0.475	0.434	0.409	0.528	0.223	0.440	0.794	0.100	0.889	1.091	1.083	0.332	0.892	0.491	0.526
Sum									2.103	2.032	1.846	2.394	2.279	1.296	2.064	9.385	0.621	10.413	9.809	9.130	1.325	8.702	5.234	4.755
Acetoc-lastic & Hydrogen-otrophic	K00577	Tetrahydrom-ethanopterin S-methyl-transferase	Subunit A	mtr	2.1.1.86	1, 3, 19	3, 19, 26	3, 19, 26	0.521	0.486	0.483	0.566	0.239	0.405	0.451	0.624	0.237	0.576	0.610	0.718	0.350	0.528	0.500	0.530
	K00578		Subunit B						0.002	0.003	0.003	0.004	0.007	0.004	0.002	0.003	0.001	0.006	0.021	0.019	0.000	0.014	0.011	0.013
	K00579		Subunit C						0.000	0.000	0.001	0.000	0.000	0.001	0.000	0.004	0.004	0.002	0.003	0.001	0.007	0.000	0.000	0.000
	K00580		Subunit D						0.063	0.053	0.046	0.084	0.024	0.052	0.067	0.040	0.032	0.023	0.005	0.013	0.068	0.016	0.018	0.027
	K00581		Subunit E						0.260	0.196	0.204	0.344	0.122	0.220	0.275	0.150	0.097	0.146	0.176	0.171	0.185	0.142	0.149	0.188
	K00582		Subunit F						0.000	0.000	0.000	0.000	0.000	0.000	0.000	0.000	0.000	0.000	0.001	0.000	0.000	0.001	0.000	0.000
	K00583		Subunit G						0.000	0.000	0.000	0.000	0.002	0.000	0.001	0.000	0.000	0.004	0.008	0.005	0.000	0.007	0.008	0.007
	K00584		Subunit H						0.246	0.245	0.260	0.394	0.229	0.222	0.233	0.633	0.199	0.713	1.209	1.470	0.299	1.086	1.002	0.841
Sum									1.093	0.984	0.996	1.392	0.624	0.905	1.029	1.455	0.570	1.471	2.034	2.397	0.909	1.792	1.688	1.606
Hydrogen-otrophic	K00200	Formylmet-hanofuran dehydrogenase	Subunit A	fmd	1.2.7.12	3, 19, 22	3, 22, 65	3, 26, 65	0.184	0.092	0.069	0.174	0.075	0.089	0.114	0.047	0.284	0.027	0.035	0.033	0.371	0.040	0.056	0.131
	K00201		Subunit B						0.289	0.308	0.280	0.338	0.077	0.250	0.250	0.095	0.444	0.107	0.137	0.150	0.444	0.142	0.166	0.246
	K00202		Subunit C						0.061	0.085	0.075	0.085	0.014	0.071	0.079	0.042	0.157	0.022	0.022	0.030	0.104	0.025	0.027	0.059
	K00203		Subunit D						0.053	0.062	0.060	0.073	0.035	0.065	0.056	0.002	0.077	0.003	0.010	0.008	0.071	0.021	0.022	0.047
	K11261		Subunit E						0.485	0.340	0.293	0.281	0.090	0.324	0.439	0.026	0.140	0.057	0.131	0.089	0.135	0.132	0.297	0.270
	K00205	Uncharacterized protein	—	—	Unknown	—	—	—	0.074	0.067	0.060	0.097	0.021	0.051	0.069	0.009	0.092	0.033	0.003	0.007	0.170	0.020	0.046	0.066
	K00672	Formylmet-hanofuran tetrahydrome-thanopterin *N*-formyltransferase	—	ftr	2.3.1.101	1, 3, 26	3, 26, 1683	3, 26, 1683	0.129	0.169	0.159	0.136	0.030	0.108	0.096	0.048	0.318	0.035	0.021	0.023	0.132	0.034	0.050	0.070
	K01499	Methenyltetra-hydromet-hanopterin cyclohydrolase	—	mch	3.5.4.27*	3, 22	3, 22	3, 22	0.047	0.067	0.050	0.081	0.015	0.052	0.032	0.004	0.074	0.011	0.020	0.022	0.145	0.026	0.021	0.054
	K00319	Methylenetetra-hydrome-thanopterin dehydrogenase	—	mtd	1.5.98.1	3, 6, 26	3, 6, 26	3, 6, 26	3.147	3.450	3.783	3.019	0.911	2.968	3.261	0.716	4.184	0.780	0.225	0.461	3.794	0.811	1.296	1.427
	K13942	5,10-methenyltetra-hydrometha-nopterin hydrogenase	—	hmd	1.12.98.2*	185	185	185	0.000	0.000	0.000	0.000	0.005	0.000	0.000	0.002	0.000	0.000	0.001	0.001	0.007	0.000	0.000	0.000
	K00320	5,10-methylen-etetrahydromethan-opterin reductase	—	mer	1.5.98.2	3, 43, 102	3, 43, 92	3, 43, 92	5.384	5.908	6.070	7.065	2.679	5.034	5.372	1.773	7.457	1.525	0.658	1.008	7.922	1.389	2.424	3.152
Sum									9.854	10.548	10.899	11.350	3.953	9.012	9.768	2.765	13.228	2.600	1.263	1.834	13.295	2.640	4.405	5.521
Common	K00399	Methyl-CoM reductase	Subunit Alpha	mcr	2.8.4.1	1, 3, 22	3, 19, 22	3, 26, 43	8.010	6.397	6.470	4.849	3.222	4.739	6.355	6.625	3.292	8.172	12.755	13.288	4.747	11.437	8.533	9.775
	K00401		Subunit Beta						8.002	6.508	6.344	4.731	1.755	4.384	5.717	5.649	4.351	4.780	6.689	6.593	6.703	5.882	5.438	5.988
	K00402		Subunit Gamma						6.186	5.383	5.367	4.618	2.532	3.885	4.731	7.039	2.708	7.129	13.444	13.935	4.923	11.247	9.987	10.420
	K03388	Hetero-disulfide reductase	Subunit A2	hdr	1.8.7.3 and 1.8.98.4–6	3, 20, 26	3, 20, 26	3, 26, 134	0.604	0.546	0.543	0.594	0.602	0.647	0.577	0.079	0.573	0.109	0.051	0.074	0.917	0.215	0.208	0.361
	K03389		Subunit B2						0.050	0.047	0.042	0.072	0.027	0.050	0.047	0.009	0.075	0.007	0.005	0.011	0.146	0.015	0.013	0.040
	K03390		Subunit C2						0.030	0.024	0.024	0.042	0.021	0.035	0.032	0.006	0.045	0.005	0.003	0.007	0.074	0.006	0.007	0.028
	K08264		Subunit D		1.8.98.1	1, 19, 22	1, 19, 22	1, 19, 22	0.003	0.002	0.002	0.032	0.022	0.001	0.018	0.213	0.001	0.180	0.157	0.232	0.004	0.142	0.123	0.096
	K08265		Subunit E						0.000	0.000	0.000	0.000	0.000	0.000	0.000	0.000	0.000	0.000	0.002	0.002	0.000	0.002	0.001	0.002
	K14126	F420-non-reducing hydrogenase	Large subunit	mvh/vhu/vhc	1.8.98.5*	3, 102, 185	3, 102	3, 102	0.035	0.037	0.044	0.022	0.025	0.021	0.018	0.004	0.028	0.035	0.009	0.015	0.048	0.023	0.025	0.018
	K14127		Iron-sulfur subunit						0.000	0.000	0.000	0.001	0.000	0.000	0.000	0.002	0.000	0.011	0.003	0.006	0.005	0.002	0.000	0.001
	K22516	Formate dehydro-genase	Subunit Alpha	fdh	1.17.98.3 and 1.8.98.6	3, 6, 26	3, 6, 26	3, 6, 26	0.392	0.660	0.663	0.665	0.355	0.474	0.538	0.558	2.457	0.431	0.153	0.301	1.524	0.263	0.248	0.425
	K00125		Subunit Beta						0.110	0.147	0.140	0.152	0.136	0.095	0.098	0.099	0.653	0.067	0.045	0.080	0.480	0.057	0.045	0.095
Sum									23.423	19.752	19.640	15.778	8.697	14.332	18.132	20.282	14.184	20.926	33.317	34.545	19.571	29.289	24.627	27.248
Methylo-trophic	K14080	Methylco-balamin: coenyzme M methylt-ransferase	—	MtaA	2.1.1.146	19, 22	19, 22	—	0.000	0.000	0.000	0.000	0.001	0.000	0.001	0.000	0.000	0.006	0.019	0.022	0.000	0.016	0.021	0.016
	K04480	Methanol-corrinoid protein *Co*-methylt-ransferase	—	MtaB	2.1.1.90	19, 22, 83	19, 83, 639	19, 22, 83	0.019	0.176	0.172	0.116	0.104	0.023	0.067	0.326	0.042	0.609	1.167	0.512	0.079	1.440	1.019	0.498
	K14081	Methanol-corrinoid protein	—	MtaC	MtaC	19, 22, 65	19, 22, 65	19, 22, 65	0.015	0.026	0.024	0.031	0.024	0.007	0.014	0.045	0.007	0.146	0.298	0.124	0.006	0.361	0.249	0.139
Sum									0.035	0.202	0.196	0.146	0.129	0.030	0.081	0.371	0.049	0.761	1.483	0.657	0.084	1.817	1.289	0.653

Shown are mean values for each plant. Number of columns E-G give species number (Supplementary Table S1) of the TOP3 organisms associated with each KEGG orthology term/enzyme class.

### Biogas plants are grouped by taxonomy and function

**Figure 2:**
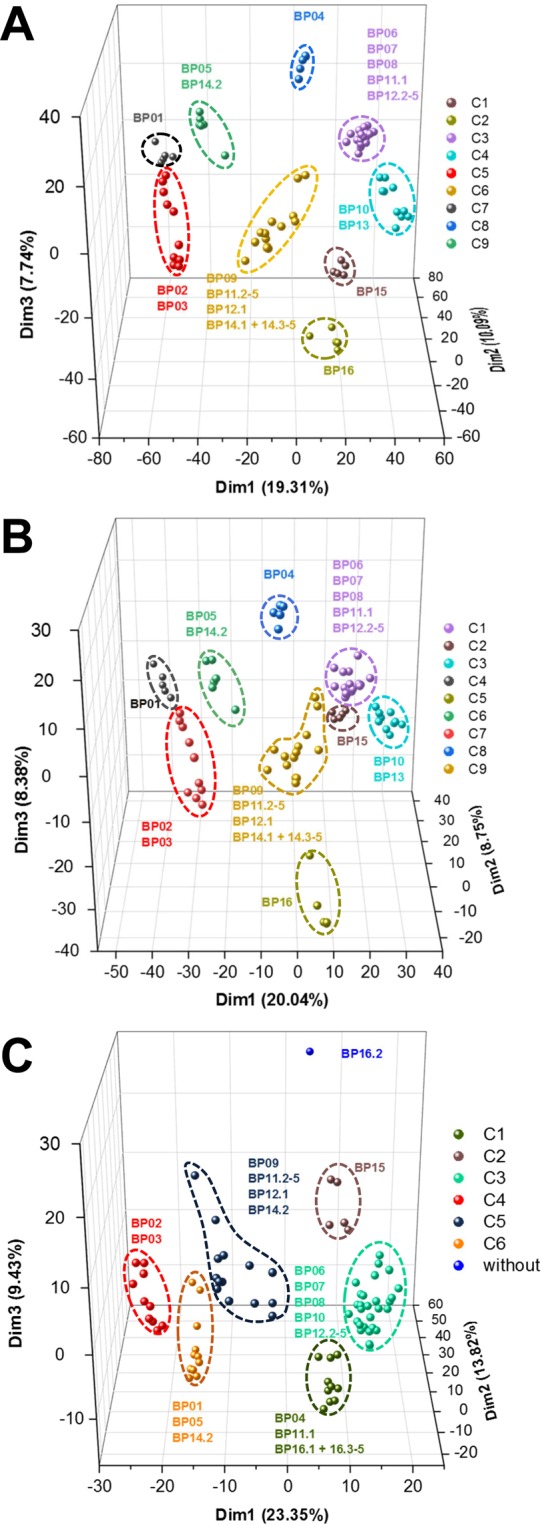
First three dimensions of principal component analysis for the different levels of proteins (**A**), species (**B**) and functions based on KEGG orthologous groups (**C**). Percentage of variance explained by each dimension is given in parentheses. C1 to C9 indicating the respective clusters according to Euclidean distance matrix.

Principal component analysis (PCA) on both, protein and species level revealed nine very similar clusters, while on functional level only six clusters were obtained (Fig. 2 and Supplementary Fig. S1). The nine different clusters (C1-9) on species level (Fig. 2B) were also found by microbial community composition analysis (Supplementary Table S1).

Bacterial families *Clostridiaceae*, *Peptococcaceae* and *Pseudomonadaceae* were prominent in all BPs (Supplementary Fig. S3). C1 (BP06 to BP08 and BP12), C2 (BP15) and C3 (BP10, BP13) additionally showed higher abundances of *Thermoanaerobacteraceae* and *Thermoanaerobacterales Family III*. C4 (BP01) was comprised with higher abundances of *Peptostreptococcaceae*, while high relative abundances for members of *Petrotogaceae* were observed in C5 (BP16). Proteins from family *Porphyromonadaceae* were particularly prominent in C6 (BP05) and C7 (BP02, BP03). Moreover, high abundances of *Enterobacteriacaeae* were present in C8 (BP04) (Supplementary Table S1).

Archaeal metaproteome profiles of C1, C3, C5 and C8 were dominated by *Methanomicrobiaceae*, and *Methanoregulaceae*. *Methanosarcinaceae* were particularly prominent in C7 and C9 (BP09, BP11 and BP14) (Supplementary Table S1).

### Process parameters drive the metaproteome profiles

Canonical correspondence analysis (CCA) indicated that temperature, pH, volatile fatty acid (VFA) concentration and different substrates (maize silage, grass silage, dry manure) significantly (p < 0.05) affected the metaproteome profiles of the respective BP (Table 2). Clusters of PCA on species level (Fig. 2B) also reflected differences in process parameters: thermophilic BPs (BP15 and BP16) were separated from remaining mesophilic BPs. In addition, biogas plants with high proportions of plant silages (e.g. C1, C3) were discriminated from BPs with high proportion of cattle slurry (C4, C7), as well as dry manure (C6). BP16 (C5) revealed high VFA concentrations (Supplementary Table S2), which was separated from BPs with lower VFA concentrations (e.g. C4, C6 and C7).

**Table 2:** Effects of environmental variables on protein profiles of the different plants.

Variable	VIF	F-value	p-value	Significance^#^
Cow manure	2.532	1.5865	0.052	
Maize silage	2.857	1.9117	0.017	*
Grass silage	4.025	1.6323	0.047	*
Corn	7.350	1.1328	0.321	
Dry manure	1.486	1.8466	0.014	*
Food residues	9.119	0.9951	0.467	
pH	3.605	1.7889	0.019	*
Temperature	3.775	2.609	0.002	**
VFA	5.101	1.6246	0.037	*
C/N ratio	4.197	0.9118	0.588	

Only parameters that showed no collinearity (variance inflation factor (VIF) < 10) were used for canonical correspondence analysis. Significance of each parameter were determined by using 9,999 permutations. #: *p ≤ 0.05, **p ≤ 0.01.

### Microbial interaction patterns are highly dependent on main methanogenic pathways

Fourteen BPs (incl. all replicates) were assigned to the same methanogenic pathway based on methanogenic protein abundances as defined by respective F-value (Supplementary Table S3). Eight BPs (BP04, BP06 to BP08, BP10, BP12, BP13 and BP16) were dominated by hydrogenotrophic methanogenesis (HyMe), while five BPs (BP01 to BP03, BP05 and BP14) were governed by acetoclastic methanogenesis (AcMe). In addition, BP09, BP11 and BP15 were assigned to both pathways (BoMe).

Number of nodes and edges differed between the networks on species level (Table 3 and Supplementary Fig. S4). HyMe had the most edges (2,260) followed by AcMe (1,984 edges) and BoNe (1,453 edges), respectively. The number of nodes were organized likewise in the same pattern (HyMe: 706, AcMe: 672 and BoMe: 504). HyMe also showed highest number of modules (42), followed by BoMe (32) and AcMe (26). Based on their topological role, nodes acting as generalists were observed for all three networks. Most of these generalistic nodes were assigned to Firmicutes, irrespective of predominant methanogenic pathway. In addition, higher proportions of generalistic nodes were assigned to Proteobacteria and Actinobacteria (HyMe and BoMe) or Euryarchaeota (AcMe). Only two nodes were acting as a generalist in more than one network. S0024 (*Peptococcaceae bacterium 1109*) was a module hub in AcMe and HyMe, while S0271 (*Defluviitoga tunisiensis*) was a module hub in HyMe and BoMe. Both species were the most abundant generalists in the corresponding networks. Remaining 46 nodes act as a generalist in only one of the networks. In AcMe and HyMe exclusively interactions between *Bacteria* and *D. tunisiensis* were observed (AcMe: 5 positive, HyMe: 10 negative), while network analysis of BoMe identified interactions (17 positive, 26 negative) between *D. tunisiensis* and different *Bacteria*, as well as different methanogens, such as *Methanosarcina barkeri* or *Methanosarcina mazei*. No direct interactions between hydrogenotrophic Archaea and known syntrophic acetate-oxidizing bacteria (SAOB) such as *Thermacetogenium phaeum* or *Syntrophaceticus schinkii* could be observed. While SAOBs showed more interactions in HyMe (*T. phaeum*: 9, *S. schinkii:* 19) and BoMe (*T. phaeum*: 4, *S. schinkii:* 3), fewer interactions were observed in AcMe (*T. phaeum*: 1, *S. schinkii:* 0).

**Table 3:** Overview of the network parameters level for acetoclastic (AcMe), hydrogenotrophic (HyMe) and both methanogenic pathways (BoMe).

Parameter		AcMe	HyMe	BoMe
Nodes		672	706	504
Edges	positive	960	1,331	903
	negative	1,024	929	550
	In-Module	1,851	2,077	1,253
	Outside-module	133	183	200
	total	1,984	2,260	1,453
No. of modules		26	42	32
Topological roles	Connector	0	5	6
	Module Hub	12	16	11
	Network Hub	0	0	0
	Peripherals	660	685	487

Affiliation of BPs to networks can be found elsewhere (Supplementary Fig. 4). Networks were calculated separately for each type of methanogenic pathway.

### *Pseudomonas* spp. carbohydrate metabolism was independent on methanogenic pathway

**Figure 3:**
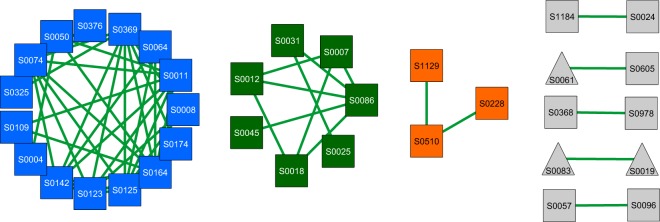
Shared nodes after merging all three networks. Only nodes with at least one common interaction throughout all networks were considered. Colors indicate group of nodes with more than one edge (blue: subgroup 1, green: subgroup 2, orange: subgroup 3, grey: remaining nodes with at least one interaction). Nodes are labeled with respective species number (Supplementary Table S1). Green color of edges correspond to positive interactions.

Further analyses of those shared nodes and edges identified, among others, 15 different *Pseudomonas spp*. acting as key players in AD for all main methanogenic pathways. They were found to share 37 edges among themselves in all networks (Fig. 3). Functional analysis revealed that they were mainly active during hydrolysis of carbohydrates using the Entner-Doudoroff (ED) pathway (Supplementary Fig. S5). Most of the high abundant proteins (e.g. succinate CoA ligase, enolase or ATP synthase) correspond to *P. fluorescens*. Expression of most protein-coding genes was consistent among all networks (Supplementary Fig. S6). Proteins upregulated in HyMe and BoMe were C3K6H6 (enolase) and A0A010SIZ3 (L-glaceraldehyde-3-phosphate reductase). In contrast, C3K613 (fatty acid oxidation complex subunit alpha) was upregulated in BoMe and AcMe, while A0A085VRFO (ornithine decarboxylase), A0A176V720 (glyceraldehyde-3-phopshate dehydrogenase) and A0A098T689 (GMP-synthase) were upregulated only in AcMe.

### Methanogenic protein patterns allow assignment to one or more methanogenic pathways

**Figure 4:**
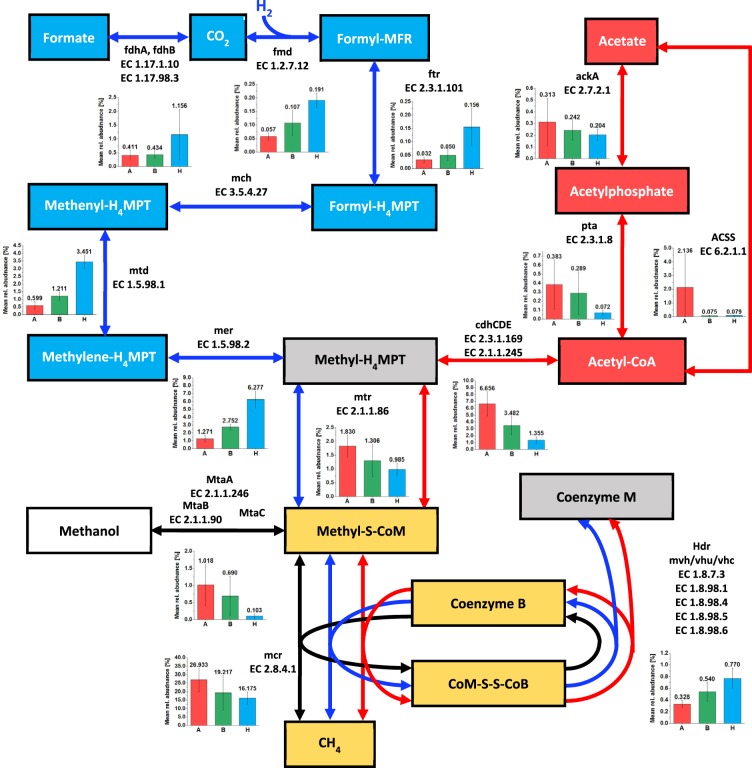
Most important steps of the three main methanogenic pathways (modified from Guo *et al*.^43^).Steps of hydrogenotrophic methanogenic pathway are colored in blue, steps of acetoclastic methanogenic pathway in red and steps of methylotrophic methanogenesis in black. Additionally, steps being part of hydrogenotrophic and acetoclastic methanogenesis are colored in grey, while steps being part of all three pathways are colored in yellow. Bar charts showing the mean relative protein abundance or each group of plants (A: acetoclastic, H: hydrogenotrophic, B: both pathways). Abbreviations: Formyl-MFR, formylmethanofuran; H4MPT, tetrahydromethanopterin; CoM, methyl-coenzyme M; CoM-SS-CoB,mixed-disulfide of coenzyme M and coenzyme B; MtaA, methylcobalamin:coenzyme Mmethyltransferase; MtaB/MtaC, methanol:5-hydroxybenzimidazolyl-cobamide Co-methyltransferase; ackA, acetate kinase; pta, phosphate acetyltransferase; ACSS, acetyl-CoA synthetase; cdhCDE, acetyl-CoA decarbonylase/synthase; fdhAB, format dehydrogenase; fmd, formylmethanofuran dehydrogenase; ftr, formylmethanofuran-tetrahydromethanopterin N-formyltransferase; mch, methenyltetra-hydromethanopterin cyclohydrolase; mtd, methylenetetrahydromethanopterin dehydrogenase; mer, 5,10-methylenetetrahydromethanopterin reductase; mtr, tetrahydromethanopterin S-methyltransferase; hdr, heterodisulfide reductase; mcr, methyl-coenzyme M reductase.

Proteins of all methanogenic pathways were found in each BP but in very different relative abundances (Fig. 4). High protein abundances were observed for *Methanoculleus bourgensis* (especially in HyMe) and *M. barkeri* (most AcMe) in all BPs. In addition, BP05 and BP13 showed high abundances of MCR from *Methanothrix soehngenii* (Table 1). Most abundant proteins specific for HyMe (Fig. 4) were methylenetetrahydromethanopterin dehydrogenase, mainly from *M. bourgensis*, *Methanospirillum hungatei* and *Methanoculleus marisnigri*, and 5,10-methylenetetrahydromethanopterin reductase (5,10-Methylene-THMPT) from *M. bourgensis* and *Methanosphaerula palustris* (Table 1). In contrast, AcMe was governed by Acetyl-coenzyme A synthetase, mainly from *M. soehngenii*, *M. mazei* and in lower abundances from *M. acetivorans*. In addition, subunits of acetyl-CoA decarbonylase/synthase complex were abundant, which were mainly affiliated to *M. soehngenii* and *M. mazei*. Higher abundances for those proteins were also observed for SAOB *S. schinkii* (Table 1). Co-methyltransferase was the only representative protein for methylotrophic methanogenesis with high abundances in few BPs (BP02, BP14 and BP09), which was mainly affiliated to *M. mazei* and *Methanosarcina sp. Ant1*.

### Link between process parameters, taxonomy and microbial functions

Process temperature was the most important driver for the metaproteomic profiles (Table 2). Members of the family *Porphyromonadaceae* were negatively correlated to temperature and families *Synergistaceae* and *Petrotogaceae* were positively correlated (Supplementary Table S4). Correlation of *Petrotogaceae* was mainly linked to thermophilic *D. tunisiensis* and its Glyceraldehyde-3-phosphatase dehydrogenase, as well as pyruvate-phosphate dikinase. No correlation of temperature and Archaea was observed.

Similarly, bacterial proteins (carbohydrate metabolism and transporter proteins) of *Peptococcaceae*, *Ruminococcaceae* and *Tissierellaceae* were positively correlated with pH, but no archaeal proteins (Supplementary Table S4). Proteins from family *Methanomicrobiaceae* (mainly *M. bourgensis*) were positively and from *Methanosaetaceae* and *Methanocellaceae* (mainly MCR subunits of *M. soehngenii, Methanosaeta harundinacea* and *M. mazei*) were negatively correlated to VFA concentrations.

## Discussion

We observed for almost all metabolic steps of AD high protein abundances for transport, glycolysis/gluconeogenesis and methanogenesis. Proteins for all different methanogenic pathways were present in each BP, indicating that a mixture of methanogenic pathways simultaneously convert agricultural substrates to biogas in large-scale BPs. Nevertheless, majority of BPs was dominated by either hydrogenotrophic or acetoclastic methanogenesis as prevalent pathway. In comparison with other approaches like stable isotope probing combined with a nucleic acid approach (DNA-SIP or RNA-SIP), metaproteomics enable accurate access to microbial phylogeny, function and its abundances in any scale of the bioreactors. In contrast, RNA- or DNA-SIP approaches are well established for small-scale bioreactors^27–30^, but expensive and artificial as results cannot easily be upscaled. Robustness and reproducibility of our approach is supported by the results for the five replicates of a BP: in only two cases one replicate was assigned to another main methanogenic pathway compared to remaining four replicates. This approach could also be attractive for plant operators. If they know the main methanogenic pathway, they could adapt relevant process parameters to optimize the AD processes in their digester to increase biogas production. For instance pH adjustment is biogas rate limiting, if hydrogenotrophic methanogenesis prevails^31^. Nevertheless, for further critical review of our approach, more metaproteome data sets and its associated process parameters from large-scale BPs have to be analyzed.

Numbers and compositions of BP clusters differed on taxonomic and functional level (Fig. 2), suggesting a highly adaptive metabolic network of various active key members in a complex microbial community. Even if similar microbial communities were present, the members fulfilled different ecological processes resulting in defined interaction patters. This in turn suggests a high grade of specialization, which is dependent on a variety of factors, such as process parameters, presence and absence of potential interaction partners or bioavailability of substrates. Therefore, to gain a preferably comprehensive understanding of the AD processes, the need for holistic approaches like metaproteomics are profitable. Results of microbial community composition on 16S rRNA gene for instance has the risk of underestimating the importance and activity of especially methanogens compared to proteomics based approaches^10^.

Environmental variables, such as substrate or temperature, are known to affect community composition during AD as revealed by nucleic acid based analyses^6,10,32^. Similarly, a variety of parameters (e.g. temperature, pH, feedstock, VFA) were found to significantly affect the metaproteome profiles of BPs what is in accordance with other studies^5,33^. Some already suggested suitable marker organisms for different types of BPs, as well as potential biomarkers for AD in BPs could be approved^5^. In addition, some potential new biomarkers were described here. In our study is *D. tunisiensis* a promising candidate for a marker organism of thermophilic BPs. This species is known to be a key player for hydrolysis in BPs, and its genome encode a variety of genes associated with complex polysaccharide degradation^34^. Positive correlations of glycolytic proteins for *D. tunisiensis* indicate a high metabolic activity of *Petrotogaceae* during degradation of complex carbohydrates under thermophilic conditions, as observed in other studies^35^.

*Peptococcaceae bacterium* 1109 was highly abundant in most BPs and positively correlated to pH. Therefore, a decrease in pH (as observed during acidification) could possibly be detected by a decrease of proteins from this species. In addition, observed correlations of pH and different species were solely positive. This is surprising as all BPs were single-stage fermenters and negative correlations between hydrolyzing organisms and pH were expected, due to their lower pH optima. This findings indicate a highly adapted bacterial community, which is able to hydrolyze substrates efficiently even at high pH ≥ 7.7 (see Supplementary Table S2) and possibly overcome bottlenecks during rate-limiting hydrolysis. Many different pathways seem to be influenced by VFA concentration (Supplementary Table S4), which is in line with previous results as high VFA concentrations inhibit both, hydrolysis and methanogenesis^36^. Nevertheless, it has been reported that high acetate (i.e. about 2400 mg/L) and butyrate concentrations (about 1,800 mg/L) seem to have no effect on methanogenesis, while high propionate concentrations (900 mg/L) are more critical^37^. Higher proportions of propionate have been observed for three BPs (BP 10, BP 12 and BP 16), that were assigned to HyMe. Although there are more hydrogenotrophic BPs, it is noticeable that these three BPs showed lower abundances of known SAOB, such as *T. phaeum* or *S. schinkii* (Supplementary Table S1). Acetate oxidation by syntrophs can be a rate limiting step of hydrogenotrophic methanogenesis^38^, and therefore a lack of SAOB during hydrogenotrophic methanogenesis is likely to lead to an accumulation of VFAs, which in turn can inhibit methanogenic activities. Therefore, monitoring the abundance of proteins from SAOBs seems to be a suitable way to check the stability and performance of hydrogenotrophic BPs. In addition, our results suggest that MCR from *Methanosarcinales* is as promising biomarker candidate for acidification. Acetoclastic organisms in combination with the presence of mixed acid fermentation enabled a fast metabolization of different VFAs. Acetoclastic microorganisms have to metabolize more substrate to obtain the same energy as hydrogenotrophic microorganisms^39,40^. As differences in process parameters as well as used substrates could also strongly influence VFA concentrations^41,42^, future studies should evaluate whether VFA monitoring is useful for all types of BPs.

Network analyses approach revealed distinct metabolic and microbial interaction patterns for each methanogenic pathway. Different number of nodes and edges for each network indicated highly complex microbial interactions patterns, with HyMe being the most complex one. In contrast, BoNe showed the fewest number of nodes and edges and could be considered to be the least complex network. Possibly, the prevalence of two methanogenesis pathways prevent the organisms from a too deep specialization and consequently, less organisms and interactions are necessary for a stable AD process. In addition, network analyses support the importance of *Defluviitoga tunisiensis*, as well as *P. bacterium* 1109 for AD. As no common interactions of both species with other microorganisms could be observed among all networks, it can be assumed that their high flexibility for different interaction partners, may lead to their generalistic behavior. In contrast, highly specialized organisms can substantially contribute to AD such as 15 different *Pseudomonas spp*., which shared 37 edges among themselves over all networks and BPs. Stable expression for most proteins of the different *Pseudomonas spp*. (Supplementary Fig. S6), indicating a highly conserved carbohydrate metabolism of those species, even if process parameters are different (Supplementary Fig. S2). Additionally, *Pseudomonas spp*. seems to prefer the Entner-Doudoroff (ED) pathway instead of the Embden-Meyerhof (EM) pathway (Supplementary Fig. S5) for glucose degradation, even if ED is energetically unfavorable. This finding may be linked to the lack of phosphofructokinase for most *Pseudomonas spp*., which is the key enzyme of EM^43^. Different *Pseudomonas spp*. metabolize glucose through ED which have been previously shown in lab based experiments of glucose metabolism^44,45^. Results of this study can be used to optimize process conditions to facilitate metabolic activity of *Pseudomonas spp*. in agricultural BPs. Even if no direct interactions between SAOBs and hydrogenotrophic methanogens could be observed, the numbers of interactions of SAOBs in each of the three networks clearly indicate the great importance of SAOBs for HyMe, while they seem to play a minor role during AcMe. During AcMe a big proportion of produced acetate is metabolized by acetoclastic methanogens, and therefore SAOBs have to compete with them. These findings were supported by higher mean protein abundances for *T. phaeum* (AcMe: 0.2%, HyMe: 1.0%, BoMe: 0.6%), as well as *S. schinkii* (AcMe: 0.3%, HyMe: 4.7%, BoMe: 1.8%).

## Conclusions

Metaproteome analyses of 16 agricultural large-scale BPs enable a deeper insight into AD. Temperature, pH, substrate and VFA concentration were identified as main drivers for metaproteomic profiles. Comprehensive correlation analyses enabled the identification of potential marker organisms for defined process conditions, such as *Petrotogaceae* for high temperatures. Moreover, monitoring the MCR of *Methanosarcinales* could be a suitable biomarker to recognize ongoing acidification and avoid process failures.

BPs clustered similar on both, species and protein level but differed on functional level, indicating a high resilience and flexibility of the microbial community. BPs were classified to acetoclastic, hydrogenotrophic or a mixture of both pathways, while methylotrophic methanogenesis was of minor importance. Such classifications could be meaningful for upcoming studies.

Network analysis of each methanogenic pathway revealed that microbial interaction patterns widely differed among BPs reflecting large differences in metabolic processes in the prevalent methanogenic pathway. However, all methanogenic pathways have in common that *Pseudomonas spp*. were main drivers in hydrolytic processes indicating their versatile metabolism in wide-ranging process conditions and substrate variabilities. Although no interactions between hydrogenotrophic methanogens and SAOBs were observed in the networks, our data emphasize the importance of syntrophic acetate-oxidation for hydrogenotrophic methanogenesis. In addition, network analysis underline the role of *D. tunisiensis* and *P. bacterium 1109* during AD of various agricultural substrates. Further research is required to obtain a deeper understanding of anaerobic degradation and to include this knowledge in control technology to make biogas production more flexible and efficient.

## Material and Methods

### Biogas plant sampling

Primary digesters of 16 large-scale BPs were sampled in Northern Bavaria in August 2016 as described previously^10^. Briefly, digester content was mixed thoroughly and all pipelines were flushed prior to sampling. Finally, approximately 300 mL of digester sludge were obtained from each BP and subsequently shock-frozen in liquid nitrogen. Samples were thereafter transferred in liquid nitrogen and stored at −80 °C for further investigations. Some process and plant parameters were collected from the plant operators, i.e. process temperature, pH, as well as types of main and additional substrates.

### Determination of VFA concentration, VFA/TAC and C, N, S content

For determination of total volatile fatty acids (VFA) content and ratio of VFAs and alkalinity (VFA/TAC), samples were centrifuged for 15 min and 4,696 × g at 4 °C. 10 mL of the liquid phase were then titrated with 0.05 M sulfuric acid (Carl Roth GmbH, Karlsruhe, Germany) to pH values of 5.00, 4.40, 4.30 and 4.00. VFA as well as VFA/TAC were measured in triplicates and calculated as described elsewhere^46^. Composition of VFA was analyzed by atres Analytik (München, Germany) using an in-house gaschromatographic approach. Contents of following VFAs were measured: acetic acid, propionic acid, isobutyric acid, butyric acid, isovaleric acid, valeric acid, hexanoic acid and heptanoic acid.

For analyzing elemental composition (C, N, S) samples were dried at 50 °C for 48 h. Afterwards samples were grinded using mortar and pestle. 10 mg of each sample were oxidized in the combustion tube of the elemental analyzer Vario EL II (Elementar Analysensysteme GmbH, Langenselbold, Germany) at 1,150 °C according to manufacturer’s instructions. Sulphanilic acid (Sigma-Aldrich Chemie GmbH, Taufkirchen, Germany) was used as standard. All analyses were performed in triplicate measurements.

### Protein extraction and sample preparation for mass spectrometric analysis

Proteins were extracted according to Heyer and colleagues with modifications^33^. Shortly, 0.5 g biomass were mixed with 300 µL of a 2 M sucrose solution (Carl Roth GmbH, Karlsruhe, Germany), 800 µL of liquid phenol (Carl Roth GmbH, Karlsruhe, Germany) and 1 g silica beads with a diameter of 0.7 µm (Carl Roth). Samples were homogenized at 5 ms^-1^ for two minutes using a ball mill (FastPrep®-24, MP Biomedicals, CA, USA), followed by centrifugation for 10 min at 10,000 × *g* for phase separation. 333 µL of the upper phenol phase, containing the proteins, were transferred and mixed with the same volume of a 1 M sucrose solution. After incubation for 10 min at 1,000 rpm, samples were centrifuged for complete phase separation using same conditions as mentioned above. 150 µL of the upper phenol phase were then mixed with fivefold volume of 0.1 M ammonium acetate (Merck KGaA, Darmstadt, Germany) solution in methanol (Carl Roth) and stored for one hour at −20 °C for protein precipitation. After precipitation, samples were centrifuged for 20 min at 10,000 × *g* and 4 °C. Protein pellets were washed twice with 500 µL of 80% acetone (Carl Roth) and 70% ethanol (Carl Roth). Each washing step was followed by incubation at −20 °C for 15 min and subsequent centrifugation for 10 min at 12,000 × *g* and 4 °C. Pellets were resuspended in 500 µL of a resuspension solution containing 7 M urea (Merck KGaA, Darmstadt, Germany), 2 M thiourea (Carl Roth) and 0.01 g mL^−1^ dithiothreitol (Carl Roth). Five independent replicates were taken from each of the 16 BPs, resulting in a total number of 80 metaproteome measurements.

Protein concentrations were determined using amido black assay modified from Schweikl *et al*.^47^. Briefly, 150 µL of amido black dye solution (0.26 mg mL^−1^ dissolved in 10% acetic acid and 90% methanol (all from Carl Roth)) was added to 25 µL of protein solution. After 5 min of incubation at room temperature, samples were centrifuged for 10 min at 16,000 × *g*. Supernatant was discarded, and 250 µL of washing solution, containing 10% acetic acid and 90% methanol, were added. Samples were centrifuged as mentioned above. This step was repeated twice. Protein pellet was thereafter resuspended in 175 µL of 0.1 M sodium hydroxide solution (Carl Roth). Protein concentration was determined by measuring absorbance at 615 nm using µDrop-plate (Thermo Fisher Scientific, Waltham, USA) and bovine serum albumin (Carl Roth) as calibration standard according to manufacturer’s instructions.

For each sample, the volume containing 50 µg of protein were mixed with fivefold the volume of absolute acetone and incubated for one hour at −20 °C. After incubation, samples were centrifuged for 20 min at 12,000 × *g* at room temperature and supernatant was discarded. Remaining protein pellets were then dried for one hour at 35 °C using a rotary evaporator. Pellets were resuspended in denaturing buffer (7 M urea, 2 M thiourea and 0.01 g/mL dithiothreitol) and mixed with Roti®-Load 1 (Carl Roth). Polyacrylamide gel electrophoresis was carried out with a 12% acrylamide separating gel and a 4% stacking gel for seven minutes at 200 V. Protein bands were visualized by using Rotiphorese^®^ Blue R (Carl Roth). After destaining, gels were scanned with Molecular Imager® Gel Doc™ XR + (Bio-Rad, Hercules, USA).

All proteins were excised as one band with a length of approximately 10 mm from the stained gel lanes. Each piece was cutted into six to eight smaller slices. All slices of a sample were transferred to a new 1.5 mL reaction tube for subsequent tryptic digestion modified from Shevchenko *et al*.^48^. Peptide lysates were completely dried for about 90 min at 35 °C. Samples were desalted using C18-ZipTips (Sigma-Aldrich Chemie GmbH, Taufkirchen, Germany) following the manufacturer instructions.

### Mass spectrometric analysis

Peptide lysates were dissolved in 0.1% formic acid prior to liquid chromatography mass spectrometry analysis (nanoLC-MS/MS). Peptide lysates (5 µL) were first loaded on the pre-column (µ-precolumn, Acclaim PepMap, 75 µm inner diameter, 2 cm, C18, Thermo Scientific) for 5 minutes, at 4% mobile phase B (80% acetonitrile in nanopure water with 0.08% formic acid) and 96% mobile phase A (nanopure water with 0.1% formic acid), and then eluted from the analytical column (PepMap Acclaim C18 LC Column, 25 cm, 3 µm particle size, Thermo Scientific) over a 150-min linear gradient of mobile phase B (4–55% B).

Mass spectrometric analysis was performed on a Q Exactive HF mass spectrometer (Thermo Fisher Scientific, Waltham, MA, USA) with a TriVersa NanoMate (Advion, Ltd., Harlow, UK) source in LC-chip coupling mode as described elsewhere^49^. Briefly, the mass spectrometer was set on loop count of 20 using for MS/MS scans with higher energy collision dissociation (HCD) at normalized collision energy of 30%. MS scans were measured at a resolution of 120,000 in the scan range of 350–1,600 m/z. MS ion count target was set to 3 × 10^6^ at an injection time of 80 ms. Ions for MS/MS scans were isolated in the quadrupole with an isolation window of 1.6 Da and were measured with a resolution of 15,000 in the scan range of 200–2,000 m/z. The dynamic exclusion duration was set to 30 s with a 10 ppm tolerance. Automatic gain control target was set to 2 × 10^5^ with an injection time of 120 ms using the underfill ratio of 1%.

Proteome Discoverer (v2.2, Thermo Scientific) was used for protein identification and the MS/MS spectra acquired were searched with Sequest HT against the protein-coding sequences of Bacteria and Archaea of the UniProt database (release 08/2018). Enzyme specificity was selected as trypsin with up to two missed cleavages allowed, using 10 ppm peptide ion tolerance and 0.05 Da MS/MS tolerances. Oxidation at methionines as the variable modifications and carbamidomethylation at cysteines as the static modification were selected. Only peptides with a false discovery rate (FDR) < 1% calculated by Percolator^50^ were considered as identified. Identified proteins were grouped by applying the strict parsimony principle, in which protein hits were reported as the minimum set that accounts for all observable peptides. Protein abundances were calculated based on the top3 approach implemented in Proteome Discoverer v2.3.

### Statistical data analysis

Proteins that were not observed in at least three out of five biological replicates of a BP were excluded from further data analyses. Principal component analysis (PCA) was done on the log2-median transformed protein intensities using the *FactoMineR* package^51^ in R. Cluster analysis was based on the Euclidian distance between the BPs (using first, second and third dimension of PCA) using the function *hclust* included in the package fastcluster^52^ with the ward.D2-method.

Effects of environmental variables (Supplementary Table S1) on protein profiles were evaluated using CCA using function *cca* of R-package *vegan*^53^. Statistical significance was calculated using an ANOVA-like permutation test with 9,999 permutations included in the same package. Only variables with a variance inflation factor smaller than 10 were considered for CCA^54^. Significant correlations of different taxonomic and functional levels with plant and process parameters were analyzed by spearman´s rho with a significance level of p ≤ 0.01 using R-package *psych*^55^. To reduce data complexity only entries of the appropriate level with a minimum mean relative abundance of at least 0.1% were used. Differences in protein abundance levels were calculated using R-package *limma*^56^.

### Determination of dominant methanogenic pathway

Mean values of protein abundances of each BP were normalized to 100%. Each protein group was assigned to the corresponding KEGG orthologous (KO) group^25^. A list of KO groups being unique for different methanogenic pathways (KEGG module 00567: hydrogenotrophic methanogenesis, KEGG module 00357: acetoclastic methanogenesis and KEGG module 00356: methanogenesis from methanol) was complied. All proteins were assigned to their corresponding Enzyme Commission number (EC)^57^. Protein abundances of all KO-Terms unique for acetoclastic and hydrogenotrophic methanogenesis were summed up. A factor F was generated by dividing the relative abundance of acetoclastic proteins through the relative abundance of hydrogenotrophic proteins. All samples with F ≥ 2.5 were assumed mainly acetoclastic, while F ≤ 0.4 indicates mainly hydrogenotrophic methanogenesis. F values between those values suggest that both pathways contribute substantially to methanogenesis.

### Network analyses

Network analyses were carried out separately for each methanogenic pathway using Cytoscape plugin CoNet^22^. To reduce data complexity, relative abundances of all proteins were summarized up to species level and a unique identifier was given for each species (Supplementary Table 2). For more valid network calculation, no mean values for each BP were calculated, but factor F was generated for each replicate. Based on their dominant methanogenic pathway, 40 replicates were used as input for the HyMe, while 25 replicates were used for AcMe calculation. Network for replicates with no clearly dominant methanogenic pathway (BoMe) was based on remaining 15 replicates. Network calculation itself was carried out with following parameters: Pearson, Kendall and Spearman correlation, as well as Bray-Curtis and Kullback-Leibler dissimilarity as methods. Minimal occurrence of observations for each set of replicates was set to at least 60% (i.e. 24, 15 and 9 observations for the different networks). Threshold was set to 2,500 top and bottom edges, so that each correlation and dissimilarity method contributed 2,500 positive and 2,500 negative edges to the initial network. MinSupport was selected to be three, so only edges supported by at least three of the five methods were kept. The method-specific p-values were computed by using the mean and standard deviation of the bootstrap distribution (100 iterations) as a parameter of the normal distribution. Method-specific p-values were then merged using the method of Brown^58^. Only edges with p < 0.05 were kept after multiple-testing correction of Benjamini and Hochberg^59^. Nodes of the final networks were assigned to modules by using GLay community algorithm^60^. Finally, within-module connectivity (z) and among-module connectivity (P_i_) were calculated as described by Guimera and Amaral^61^ with an automated in-house excel sheet. Peripheral nodes (specialists) were defined by z ≤ 2.5 and P_i_ ≤ 0.62, connectors by z ≤ 2.5 and P_i_ > 0.62, module hubs by z > 2.5 and P_i_ ≤ 0.62 and network hubs by z > 2.5 and P_i_ > 0.

## Supplementary information

Supplementary Dataset

Supplementary Table S1

Supplementary Table S3

Supplementary Table S4

## Data Availability

The datasets generated during and/or analysed during the current study are available in the PRIDE repository, with the dataset identifier PXD014605.
