# Supplementary material for: Pseudomonas spp. are key players in agricultural biogas substrate degradation
Source: Sci Rep. 2019 Sep 6;9:12871. doi: 10.1038/s41598-019-49313-8 (PMC6731289; doi:10.1038/s41598-019-49313-8)
Supplement: Supplementary file 1 — Supplementary Dataset [file 41598_2019_49313_MOESM1_ESM.docx]

**Supplementary Material**

***Pseudomonas spp.* are key players in agricultural biogas substrate degradation**

Christian Buettner^1^, Martin von Bergen^2,3^, Nico Jehmlich^2^ & Matthias Noll^1,*^

^1^Coburg University of Applied Sciences and Arts, Institute for Bioanalysis, Friedrich-Streib-Str. 2, 96450 Coburg, Germany

^2^Helmholtz-Centre for Environmental Research – UFZ GmbH, Department of Molecular Systems Biology, Permoserstr. 15, 04318 Leipzig, Germany

**^3^University of Leipzig, Institute for Biochemistry,** Brüderstraße 34, 04103 Leipzig, Germany

**Supplementary Figures**

**
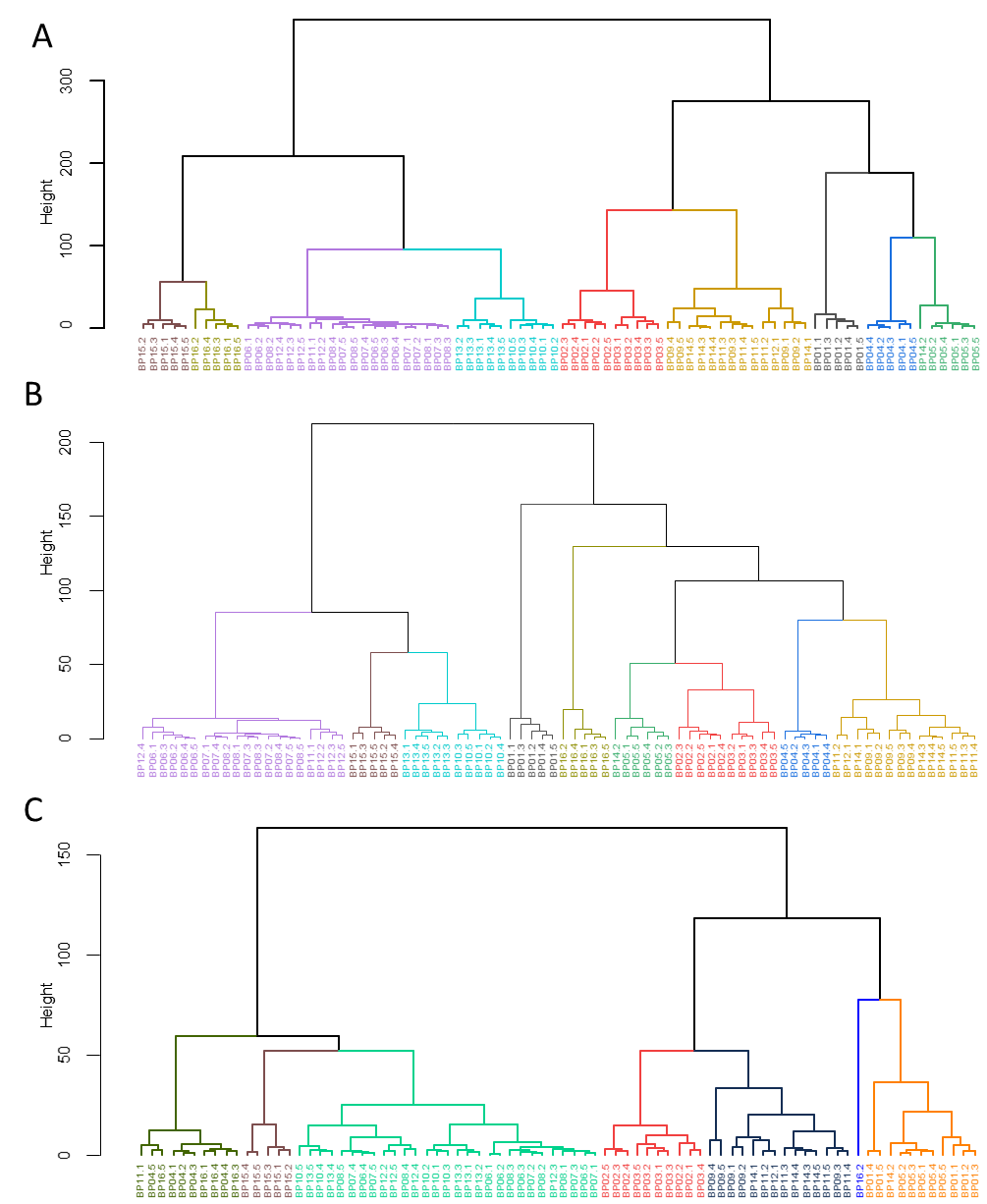
**

**Supplementary Figure S1.** Clustering of the different principal component analyses (Figure 2) on protein (A), species (B) and KEGG term level (C). Dendrograms are based on Ward.D2 distances of the samples in the first three dimensions. For clustering, a height of 50 was chosen for each dendrogram. Colors are in line with clusters in figure 2.


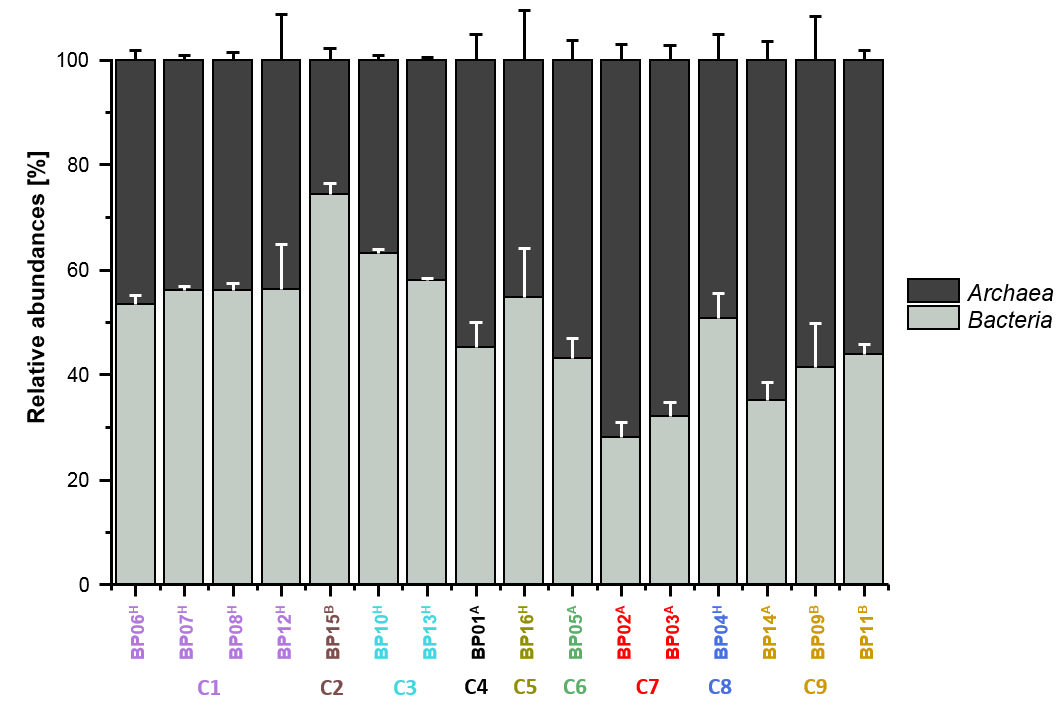


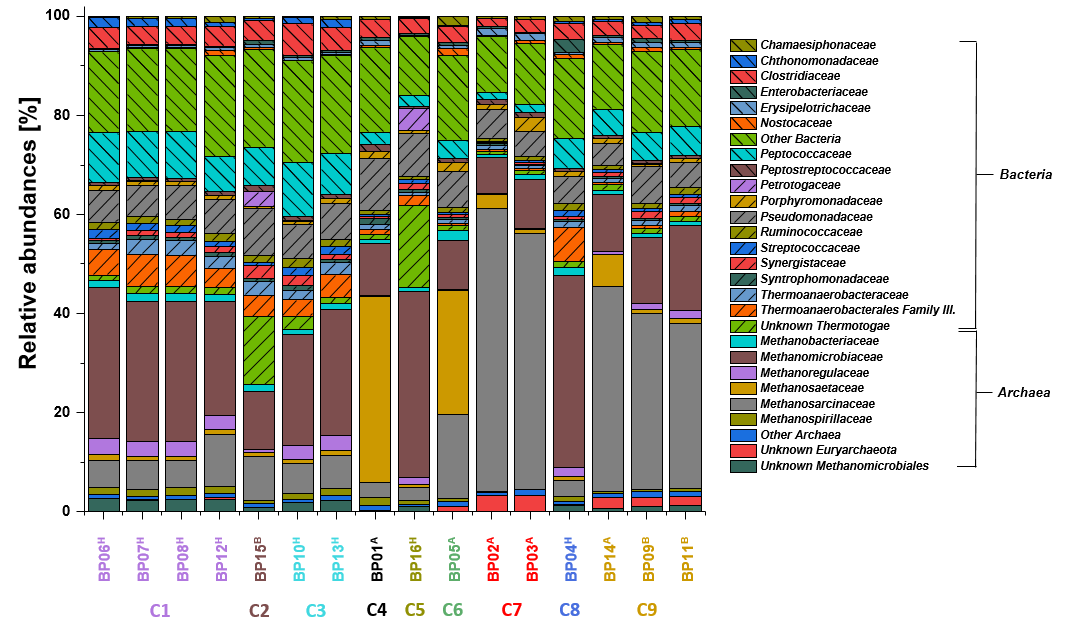


**Supplementary Figure S2.** Relative abundances of bacterial and archaeal proteins for each plant. Standard deviation is given for the five replicates of a plant. Superscripted letters state main type of methanogenesis (A: acetoclastic, H: hydrogenotrophic, B: both pathways). Color of plant label refers to clusters of figure 2B.

**Supplementary Figure S3.** Microbial Community composition for each plant on family level. Shown are mean relative abundances for each plant based on five replicates. Superscripted letters state type of main methanogenesis pathway (A: acetoclastic; H: hydrogenotrophic, B: both pathways). Color of plant label refers to clusters of figure 2B. Community compositions for other taxonomic levels can be found elsewhere (Supplementary Table S1).


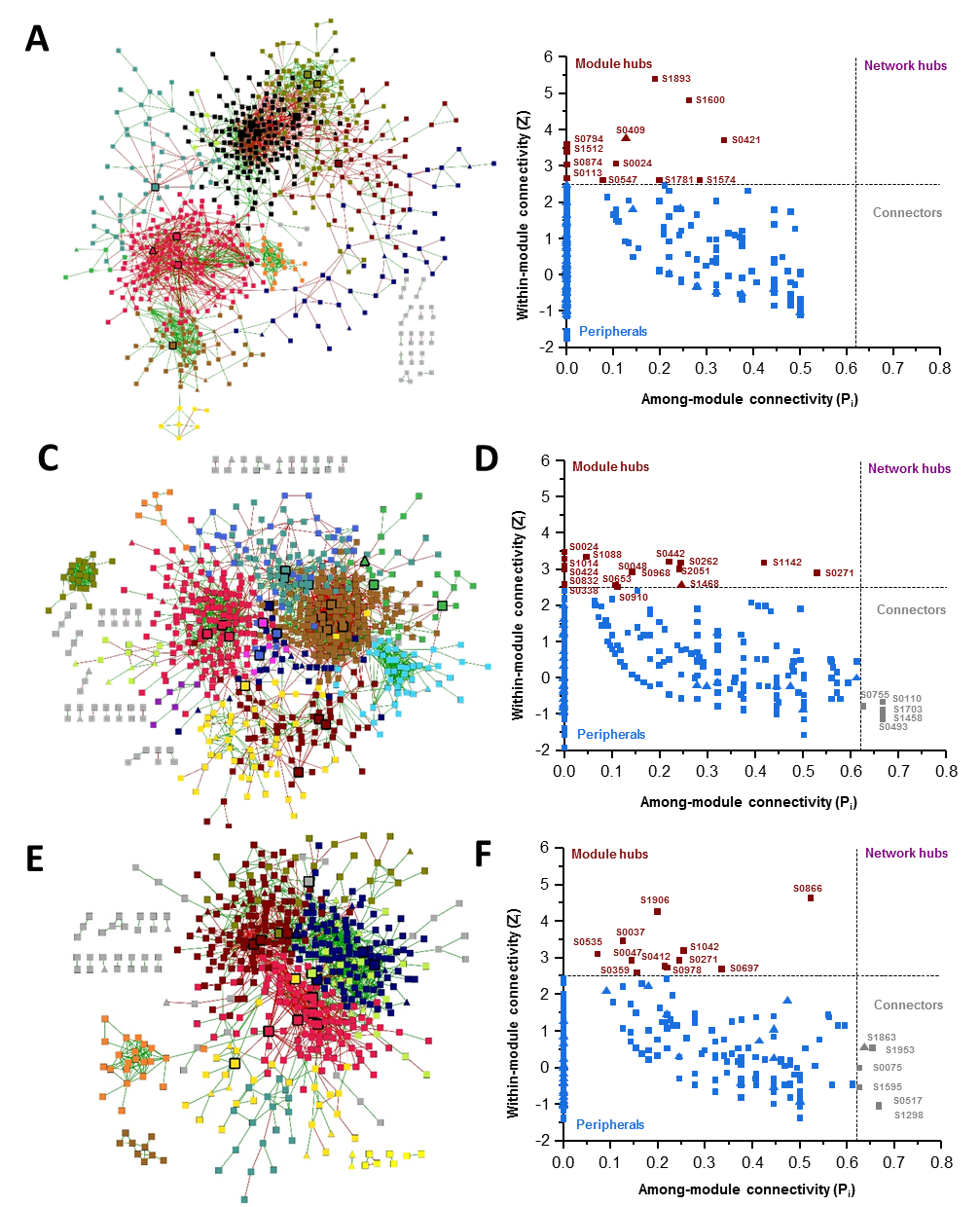


**Supplementary Figure S4.** Calculated networks on species level (A, C, E) with corresponding Z-P plots (B, D, F) according to main methanogenesis pathway (acetoclastic AcMe: A, B; hydrogenotrophic HyMe: C, D; both BoMe: E, F). *Bacteria* are denoted by squares and *Archaea* by triangles, respectively. In the networks, all nodes acting as generalists are bigger in size and additionally marked by a black frame. All nodes in the Z-P plots, which are acting as a generalist (module hubs, network hubs, connectors) are marked with the corresponding species number (Supplementary Table S1). Node color in the networks is chosen according to their module in the respective network. Green edges indicate positive and red edges negative interactions.


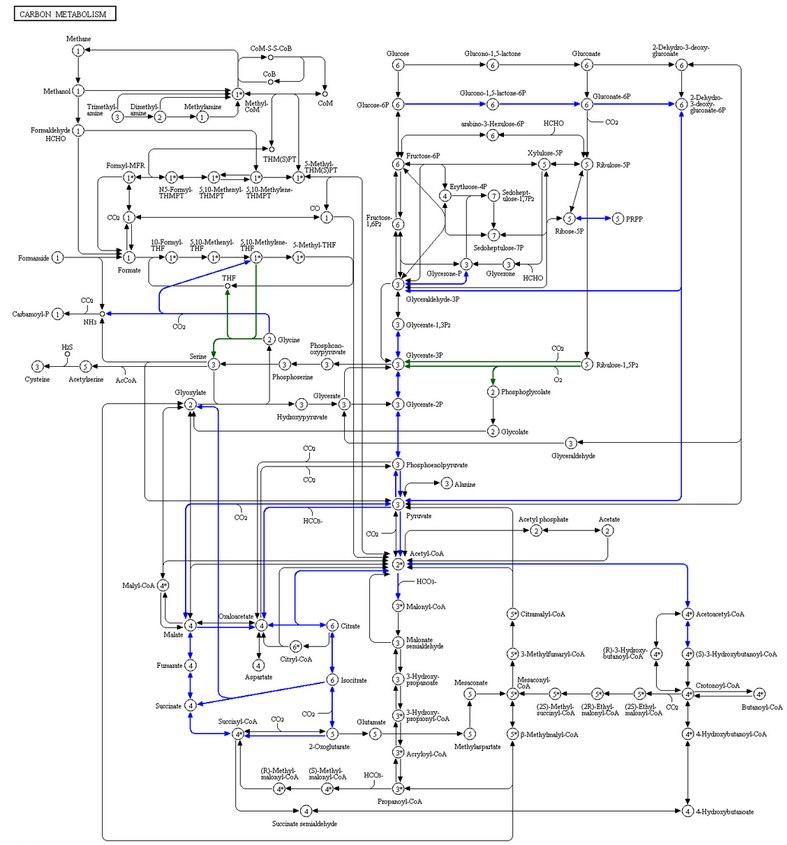


**Supplementary Figure S5.** Protein mapping of the two bigger subgroups of Figure 3 to the central carbon metabolism of KEGG^1-3^. Proteins of subgroup 1 are colored in blue, while proteins of organisms of subgroup 2 are colored in green.

**
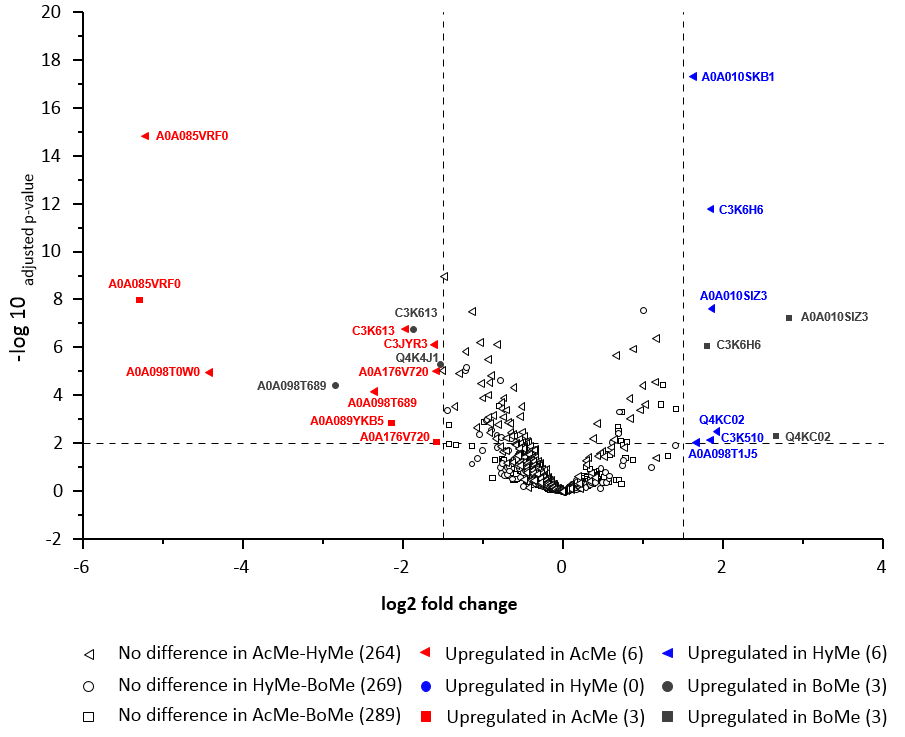
**

**Supplementary Figure S6.** Volcano plot for differential protein expression of different *Pseudomonas spp*. (Supplementary Figure S5) for all main methanogenesis pathways. Differential expression was calculated separately for each comparison (AcMe-HyMe: ; HyMe-BoMe: ; AcMe-BoMe: ). Differential expressed proteins are filled with an appropriate color and labeled with respective Uniprot-ID (Supplementary Table S1). Resulting p-values were adjusted according to Benjamini and Hochberg.

**Supplementary Tables (Excel-files are marked with*)**

**Supplementary Table S1*.** Community composition of each plant on different taxonomic levels. Given is the mean value for the five replicates of a plant.

**Supplementary Table S2.** Overview of plant and process parameters. All parameters marked with a star showed a low variance inflation factor (VIF < 10) and have therefore been part of the canonical correspondence analysis (Table 2). For triplicate measurements, the standard deviation is given.

| **Plant** | | **BP06** | **BP07** | **BP08** | **BP12** | **BP15** | **BP10** | **BP13** | **BP01** | **BP16** | **BP05** | **BP02** | **BP03** | **BP04** | **BP09** | **BP11** | **BP14** |
| --- | --- | --- | --- | --- | --- | --- | --- | --- | --- | --- | --- | --- | --- | --- | --- | --- | --- |
| **pH*** | | 8 | 7.9 | 7.7 | 7.7 | 8 | 8.1 | 7.7 | 7.5 | 7.5 | 7.9 | 7.6 | 7.7 | 7.7 | 7.8 | 7.8 | 7.7 |
| **Process temperature [°C]*** | | 43 | 43 | 43 | 45 | 54 | 45 | 45 | 38 | 60 | 42 | 38 | 42 | 42 | 45 | 45 | 45 |
| **Substrates* [%]** | **Cattle slurry** | 0 | 30 | 30 | 40 | 48 | 35 | 30 | 83 | 0 | 0 | 78 | 85 | 32 | 65 | 35 | 45 |
|  | **Maize silage** | 80 | 35 | 35 | 35 | 32 | 20 | 40 | 1 | 0 | 35 | 0 | 0 | 44 | 25 | 55 | 35 |
|  | **Grass silage** | 0 | 15 | 15 | 15 | 2 | 0 | 20 | 0 | 0 | 28 | 0 | 0 | 16 | 10 | 5 | 10 |
|  | **Corn** | 20 | 20 | 20 | 5 | 15 | 45 | 10 | 0 | 0 | 2 | 0 | 15 | 6 | 0 | 5 | 10 |
|  | **Dry manure** | 0 | 0 | 0 | 5 | 3 | 0 | 0 | 6 | 0 | 35 | 0 | 0 | 2 | 0 | 0 | 0 |
|  | **Food residues** | 0 | 0 | 0 | 0 | 0 | 0 | 0 | 10 | 100 | 0 | 22 | 0 | 0 | 0 | 0 | 0 |
| **Fermentation type*** | | liquid | liquid | liquid | liquid | liquid | liquid | liquid | liquid | liquid | Dry | liquid | liquid | liquid | liquid | liquid | liquid |
| **VFA [mg/L]*** | | 1395 ± 96 | 1374 ± 36 | 1468 ± 80 | 2780 ± 163 | 1531 ± 32 | 3754 ± 353 | 1370 ± 137 | 462 ± 56 | 7837 ± 50 | 792 ± 44 | 489 ± 50 | 571 ± 1 | 1819 ± 313 | 636 ± 2 | 996 ± 2 | 820 ± 137 |
| **VFA/TA*** | | 0.16 ± 0.01 | 0.23 ± 0.01 | 0.21 ± 0.01 | 0.49 ± 0.05 | 0.26 ± 0.00 | 0.55 ± 0.02 | 0.19 ± 0.01 | 0.14 ± 0.01 | 1.44 ± 0.03 | 0.16 ± 0 | 0.12 ± 0.01 | 0.17 ± 0.01 | 0.33 ± 0.07 | 0.12 ± 0.01 | 0.16 ± 0.00 | 0.16 ± 0.03 |
| **Volatile fatty acid composition [mg/L]** | **Acetic acid** | 58 | 683 | 557 | 2064 | 796 | 1847 | 348 | <55 | 1812 | 307 | <55 | 338 | 1261 | 63 | 494 | 225 |
|  | **Propionic acid** | <40 | 84 | 78 | 1007 | 140 | 3807 | 364 | <40 | 13296 | <40 | <40 | 112 | 153 | <40 | <40 | <40 |
|  | **Isobutyric acid** | <55 | <55 | <55 | 302 | <55 | 318 | <55 | <55 | 2338 | <55 | <55 | <55 | <55 | <55 | <55 | <55 |
|  | **Butyric acid** | <40 | <40 | <40 | <40 | <40 | <40 | <40 | <40 | 78 | <40 | <40 | <40 | <40 | <40 | <40 | <40 |
|  | **Isovaleric acid** | <40 | <40 | <40 | 90 | <40 | 90 | <40 | <40 | 1311 | <40 | <40 | <40 | <40 | <40 | <40 | <40 |
|  | **Valeric acid** | <40 | <40 | <40 | <40 | <40 | <40 | <40 | <40 | 162 | <40 | <40 | <40 | <40 | <40 | <40 | <40 |
|  | **Hexanoic acid** | <55 | <55 | <55 | <55 | <55 | <55 | <55 | <55 | <55 | <55 | <55 | <55 | <55 | <55 | <55 | <55 |
|  | **Heptanoic acid** | <40 | <40 | <40 | <40 | <40 | <40 | <40 | <40 | <40 | <40 | <40 | <40 | <40 | <40 | <40 | <40 |
| **Elemental composition [%]** | **N** | 3.34 ± 0.17 | 2.89 ± 0.02 | 2.53 ± 0.06 | 3.08 ± 0.01 | 2.81 ± 0.22 | 2.86 ± 0.03 | 3.12 ± 0.21 | 3.38 ± 0.17 | 2.58 ± 0.20 | 4.03 ± 0.27 | 2.81 ± 0.09 | 3.12 ± 0.16 | 3.22 ± 0.07 | 2.36 ± 0.10 | 3.52 ± 0.11 | 2.94 ± 0.03 |
|  | **C** | 42.17 ± 1.17 | 40.05 ± 0.71 | 39.55 ± 0.66 | 42.27 ± 0.37 | 41.94 ± 0.55 | 43.75 ± 0.22 | 41.81 ± 1.08 | 39.67 ± 0.46 | 38.19 ± 0.18 | 43.72 ± 0.35 | 39.82 ± 0.46 | 39.94 ± 1.66 | 43.44 ± 1.01 | 40.03 ± 0.87 | 42.94 ± 0.87 | 39.51 ± 0.26 |
|  | **S** | 0.52 ± 0.02 | 0.58 ± 0.06 | 0.55 ± 0.04 | 0.54 ± 0.05 | 0.58 ± 0.04 | 0.49 ± 0.02 | 0.51 ± 0.04 | 0.68 ± 0.01 | 0.42 ± 0.05 | 0.69 ± 0.14 | 0.73 ± 0.04 | 0.59 ± 0.10 | 0.52 ± 0.02 | 0.56 ± 0.05 | 0.6 ± 0.02 | 0.45 ± 0.01 |
|  | **C/N*** | 12.64 ± 0.35 | 13.83 ± 0.34 | 15.64 ± 0.26 | 13.72 ± 0.11 | 14.97 ± 1.36 | 15.31 ± 0.12 | 13.45 ± 0.66 | 11.76 ± 0.56 | 14.88 ± 1.18 | 10.88 ± 0.68 | 14.17 ± 0.28 | 12.8 ± 0.59 | 13.5 ± 0.36 | 16.99 ± 0.78 | 12.21 ± 0.42 | 13.42 ± 0.15 |

**Supplementary Table S3*.** Calculation of factors F for each replicate based on relative abundances of KO groups as explained in material and methods section.

**Supplementary Table S4*.** Overview for all calculated spearman correlations. Different worksheets display various taxonomic and functional levels. Assignment for each taxonomic and functional level can be found in Supplementary Table S1.

**References**

1. Kanehisa, M., Sato, Y., Furumichi, M., Morishima, K. & Tanabe, M. New approach for understanding genome variations in KEGG. *Nucleic acids research* **47,** D590-D595; 10.1093/nar/gky962 (2019).

2. Kanehisa, M. KEGG. Kyoto Encyclopedia of Genes and Genomes. *Nucleic Acids Res.* **28,** 27–30; 10.1093/nar/28.1.27 (2000).

3. Kanehisa, M., Sato, Y., Kawashima, M., Furumichi, M. & Tanabe, M. KEGG as a reference resource for gene and protein annotation. *Nucleic Acids Res.* **44,** D457-62; 10.1093/nar/gkv1070 (2016).
